# DRAG AS PERFORMANCE, THERAPY, ACTIVISM, AND PERSONAL EXPRESSION: WHAT OLDER DRAG ARTISTS TEACH US

**DOI:** 10.1093/geroni/igae098.0744

**Published:** 2024-12-31

**Authors:** Laura Donorfio, Brian Chapman, Brian Chapman

**Affiliations:** Chair; Co-Chair; Discussant

## Abstract

Drag artists (also described as drag performers, drag queens, and drag kings) have contributed to world cultures for centuries. Drag artistry, as tied to sexual and gender minority (SGM) communities and aging, is the focus of this presentation. The 20th and 21st centuries have been a poignant time for drag artists in the United States, as they have emerged as national and community leaders, been subject to political attack and violence, and contributed to mainstream and sexual and gender minority cultures. Older adult drag artists hold rich lived experiences and collectively share in episodic events that can be seen as traumatic, unifying, and celebrative. However, this population of older adults is grossly underrepresented in the literature. This symposium includes four presentations bridging behavioral science (individual development and wellbeing), social policy, and practice (drama therapy) of older drag artists. The first presentation frames current anti-drag policies aimed broadly at SGM communities in the United States. The second presentation provides a feminist, gender, and queer theoretical lens for examining the experiences of older drag artists, particularly the topics of homophobia, and ageism. The third presentation presents findings from a mixed methods study examining the intersection of aging, drag artistry, and individual wellbeing. The final presentation details a study on coping mechanisms of older drag artists through the lens of drama therapy clinical assessment. Collectively these papers highlight theory and qualitative and quantitative analysis illuminating themes of resilience, coping, commitment, and conscientiousness that illustrate fortitude within this population of influential artists.

